# Application and progress of temperature-sensitive hydrogels in cartilage injury repair

**DOI:** 10.3389/fbioe.2025.1602303

**Published:** 2025-08-06

**Authors:** Long Yu, Kunhao She, Rui He, Qingyu Xu

**Affiliations:** ^1^ Department of Pediatric Surgery, Hong Qi Hospital Affiliated to Mudanjiang Medical University, Mudanjiang, Heilongjiang, China; ^2^ Surgical Teaching and Research Section, The First Clinical Medical College of Mudanjiang Medical University, Mudanjiang, Heilongjiang, China

**Keywords:** temperature-sensitive hydrogel, response principle, cartilage injury, regenerative medicine, clinical application

## Abstract

Articular cartilage injury is an important challenge in the field of orthopedics. Due to its unique characteristics of being vascularless, neuralless, and without lymphoid tissue, as well as the poor proliferation and migration ability of chondrocytes, the self-repair ability of cartilage after injury is limited. In recent years, with the development of tissue engineering, temperature-sensitive hydrogels, a new type of biomedical material, have unique temperature-responsive phase transition characteristics (such as a phase transition critical point close to the physiological temperature) that enable them to rapidly form a stable three-dimensional porous structure triggered by body temperature after being injected into the joint cavity. The material is injectable, will form a gel *in situ*, and can construct a dynamic bionic extracellular matrix (ECM) microenvironment. Compared with chemically cross-linked hydrogels, this material can achieve precise spatiotemporal control without introducing exogenous stimuli, significantly reducing the risk of cytotoxicity. Through adjustable mechanical properties, highly efficient loading, and release of bioactive factors, as well as viscoelastic characteristics similar to natural cartilage matrices, it has shown great potential in the repair of articular cartilage injuries. This article reviews the research progress of temperature-sensitive hydrogels in the repair of articular cartilage injuries from aspects such as biological characteristics, mechanism of action, clinical applications, and challenges faced, providing new ideas and possibilities for cartilage injury repair.

## 1 Introduction

**FIGURE 1:**
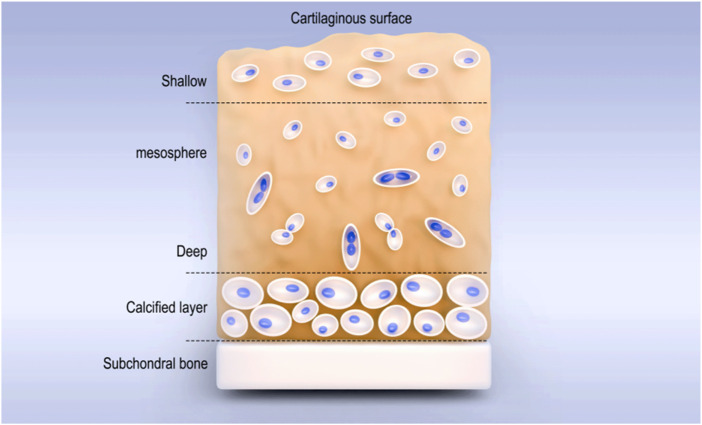
Stratification of cartilage.

Articular cartilage is a smooth and elastic connective tissue located on the surface of the articular bone and consistent in shape with the articular surface of the bone. It includes the superficial (outer) layer, the middle layer (central area), the deep layer, and the calcified area (Figure 1). The thickness of different articular cartilages depends on the content, the structure of the extracellular matrix (ECM), and the state of chondrocytes (Rahvar et al., 2024). As hyaline cartilage, its ECM is composed of type II collagen (COLII) fibers (15%–25%), proteoglycans (5%–10%), and water (5%–10%) (Li H. S. et al., 2023; Wang Z. W. et al., 2024), and it has functions such as buffering pressure and vibration, reducing friction (Karan et al., 2022), secreting synovial fluid, lubricating, protecting subchondral bone, and load transmission (Liu et al., 2024). However, due to the lack of tissues such as blood vessels, lymphatic vessels, and nerves (Song et al., 2023; Xi et al., 2023), once the articular cartilage is damaged, its self-repair ability is extremely limited. When the full-thickness cartilage is defective and the range is greater than 4 mm^3^, it cannot repair spontaneously (Benmassaoud et al., 2020). Traditional surgical methods such as drilling, microfracture, and cartilage transplantation can promote cartilage repair to a certain extent. However, the repaired tissue is often fibrocartilage, which has lower mechanical properties than hyaline cartilage and is prone to the occurrence of osteoarthritis (Sang et al., 2022; Marcus et al., 2021). Therefore, it is particularly important to find more effective methods for cartilage injury repair.

Temperature-sensitive hydrogels, a kind of intelligent responsive biomaterial, have the core advantage that the phase transition temperature can be precisely controlled to around 37°C through molecular design, and they can undergo sol–gel phase transformation at physiological temperatures. This enables the gel to precisely fill the irregular defect area through minimally invasive injection in the liquid state, and immediately transform into a solid gel under the effect of body temperature, perfectly adhering to the injured area (Jin et al., 2024) and restoring the anatomical shape. The formed three-dimensional network structure not only simulates the porosity and compression modulus of natural cartilage ECM (Kalantarnia et al., 2025) but can also guide the polar arrangement of chondrocytes through topological structure, maintain the cell phenotype, and promote the expression of COLⅡ/aggrecan genes. Compared with photocurable or pH-sensitive hydrogels, the characteristics of no initiator residue and no local pH disturbance are more in line with the physiological environment requirements of the joint cavity.

In terms of controlled drug release, the 177Lu-nucleotide coordination polymer thermosensitive hydrogel reported by Liu P. et al. (2025) achieved dual sustained release of radionuclides and anti-inflammatory factors. Real-time PET-CT monitoring confirmed that its retention time in the joint cavity was prolonged to 21 days. Integrating growth factors such as TGF-β3/BMP-2, mechanical support, and temporal regulation of biological signals can be achieved simultaneously through the temperature-controlled sustained-release system (Zou et al., 2024). The introduction of 4D printing technology enables gradient thermosensitive hydrogel scaffolds to undergo pre-programmed morphological remodeling with temperature changes. For instance, the GelMA/F127DA dual-responsive hydrogel designed by Sun et al. (2025a) can dynamically release MMP inhibitors in response to the mechanical stress generated by joint movement while triggering gelation with body temperature. These innovative achievements have laid a new technical foundation for the precise application of temperature-sensitive hydrogels in cartilage repair and have shown broad application prospects in the repair of articular cartilage injuries.

## 2 Characteristics and response principles of temperature-sensitive hydrogels

A temperature-sensitive hydrogel is a kind of soft polymer material with a three-dimensional network structure, which can quickly sense and respond to temperature changes, and has hydrophilic swelling properties (Pearce et al., 2022), biomechanical properties (Sun et al., 2025b), and plastic deformability (Mohammad et al., 2020). It is an intelligent, responsive material that can change its physical state in response to variations in internal and external environmental temperatures. In the human body, phase changes can occur along with variations in body temperature, thereby achieving precise filling and fixation of the injured area. Temperature-sensitive hydrogels not only have temperature-sensitive properties but also have good biocompatibility, degradability, and injectability, providing the possibility for the repair of articular cartilage injuries.

### 2.1 Temperature sensitivity

**FIGURE 2:**
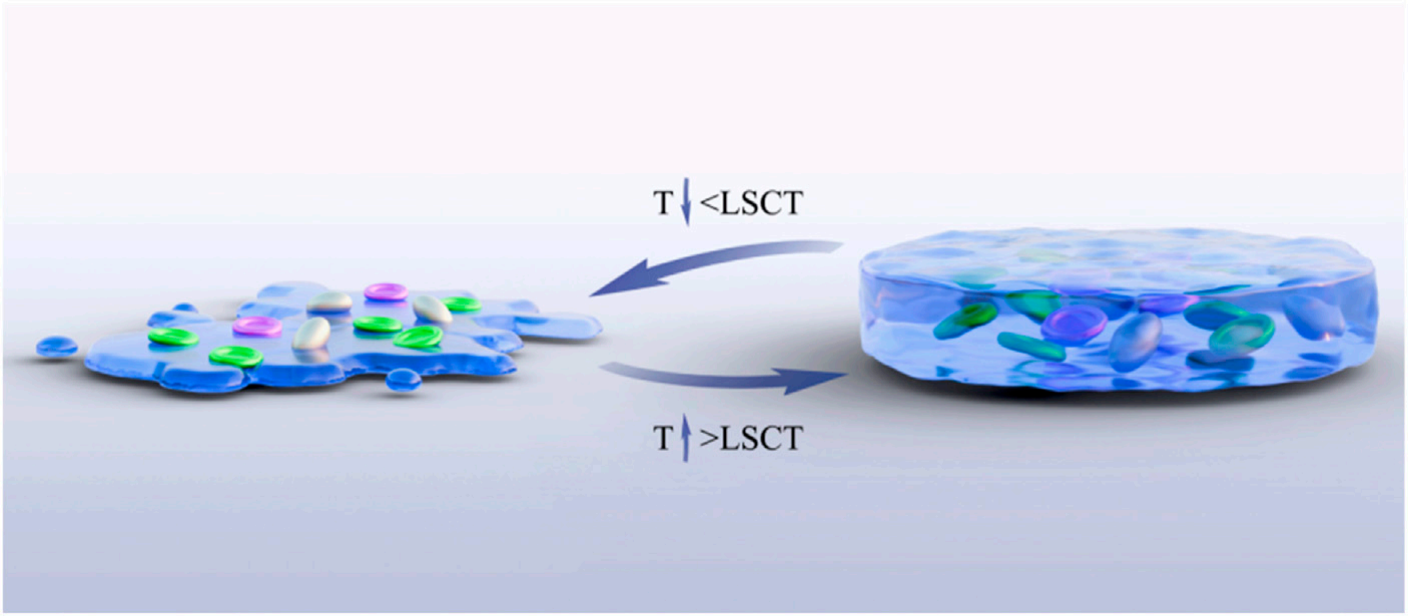
Transformation of gel phase at critical dissolution temperature.

The temperature-sensitive performance of temperature-sensitive hydrogels is one of their most significant and critical characteristics. Copolymers can undergo phase transformation through appropriate temperature changes, and this appropriate temperature is the lower critical solution temperature (LCST) (Moon et al., 2024). This temperature is usually close to the human body temperature, that is, between 32.0°C and 37.0°C. Under LCST, a reversible sol–gel phase transformation occurs (Figure 2). When the temperature is lower than LCST, it presents a sol state, with good fluidity and injectability. When the temperature increases above the LCST, the hydrogel rapidly transforms into a gel state, forming a stable three-dimensional network structure. This phase change behavior is usually caused by the hydrophilic–hydrophobic equilibrium change of the hydrogel polymer chain (Sun T. C. et al., 2024). The porous structure enables the joint to have high water content and remain moist, reducing the friction between the joints and allowing the diffusion and absorption of various solutes and nutrients (Liu et al., 2021; Liu Y. et al., 2025). Liu D. et al. (2025) developed a multifunctional temperature-sensitive hydrogel based on chitosan (CS), using chitosan (CS)/β-glycerophosphate (β-GP) as the main body of the hydrogel. The gelation transformation was achieved at physiological temperature, and the physical properties changed accordingly, promoting the repair of bone and cartilage. Han et al. (2024) confirmed through rat experiments that heterogeneous DNA hydrogels loaded with tetrahedral skeletal nucleic acids modified by Apt02 could effectively promote the injury repair of bone and cartilage in the physiological temperature environment of the human body and achieved good results. Temperature-sensitive hydrogels are classified according to the transformation type, as detailed in Table 1, and according to the source of materials, as detailed in Table 2.

**TABLE 1:** Classification of temperature-sensitive hydrogels according to the transformation type.

Transformation type	Material example	Application	*In vitro*/*in vivo* experiments	Remarks	References
Low-temperature gelation (gelation upon heating below body temperature)	Poly N-isopropyl acrylamide (PNIPAM)	Cell encapsulation, 3D bioprinted scaffolds	*In vitro*/*in vivo* experiments	The phase transition temperature is 32°C–34°C. After injection, a gel is formed at body temperature, supporting the proliferation of chondrocytes.	Guan et al. (2024), Jiang et al. (2025a), Kong et al. (2024)
	Pluronic F127 (Polosam 407)	Drug delivery (controlled release of growth factors)	*In vitro*/*in vivo* experiments	The temperature-sensitive range is 25°C–30°C, and it is often compounded with hyaluronic acid to enhance the mechanical properties.	Yang et al. (2025), Xu et al. (2024)
	Methyl cellulose (MC)	Temporary cartilage filling material	Mainly *in vitro* research	Sol-body temperature gel formation at low concentration, but the degradation rate needs to be optimized.	Hwang et al. (2024), Chen et al. (2025a), Huang et al. (2022)
High-temperature gelation (gelation close to/above body temperature)	Chitosan/β-sodium glycerophosphate complex hydrogel	*In vivo* cartilage defect repair scaffold	Mainly *in vivo* research	The phase transition temperature is 37°C–40°C. Pre-cooling of the injection is required, and it has excellent biocompatibility.	Liu et al. (2025c), Yi et al. (2023a)
	Poly (ε-caprolactone -co-lactide)-B-polyethylene glycol (PCLA-PEG-PCLA)	*In situ* gelation scaffold + stem cell delivery	*In vitro*/*in vivo* experiments	The temperature-sensitive range is 30°C–45°C, and mesenchymal stem cells (MSCs) can be loaded to promote cartilage regeneration.	Plugariu et al. (2023), Carrêlo et al. (2024)
	Hydroxypropyl methylcellulose (HPMCC)	*In vitro* cartilage tissue engineering model	Mainly *in vitro* research	It must be used in combination with light crosslinking technology to enhance stability and is suitable for *in vitro* simulation of microenvironments.	Wu et al. (2022), Zamini et al. (2025)

**TABLE 2:** Classify temperature-sensitive hydrogels according to the source of materials.

Source	Polymers	Material type	LCST(°C)	Response speed	Mechanical strength	Cytotoxicity	Key advantage	Application	References
Natural hydrogels	CS	Polysaccharide	37°C	Minute-level	Medium	Low	Antibacterial property and mucosal adhesion	Drug delivery, joint lubrication, and anti-inflammatory treatment	Maneechan et al. (2025), Vukonic et al. (2025), Wan et al. (2025)
	MC	Cellulose	32°C–40°C	Minute-level	High	Without	High mechanical strength and degradability	Controlled-drug release, 3D cell culture, and cartilage filling materials	Pan et al. (2025), Niu et al. (2025)
	Collagen	Protein	25°C–35°C	Minute-level	Medium	Without	Excellent cell adhesion and thermal reversibility	Soft tissue engineering and trauma repair	Zhang et al. (2025a), Kumar et al. (2025), Zhong et al. (2025)
Synthetic hydrogels	PNIPAAm	Polymer	32°C–34°C	Second-level	Medium - High	Low	Precise controlled release and fast response	Cell encapsulation and 3D bioprinted scaffolds	Younus et al. (2024), Xing et al. (2023)
	Poloxamer	Copolymer	25°C–30°C	Minute-level	Low- medium	Without	High biocompatibility and *in situ* injection	Drug delivery and controlled release of growth factors	Feng et al. (2021), Helena et al. (2021)
	Chitosan/β-GP	Complex	37°C–40°C	Minute-level	Minute	Low	Antibacterial and dual-responsive	Cartilage defect repair scaffold	Xu et al. (2021), Zhao et al. (2025), Liu and Ma (2025)

### 2.2 Biocompatibility

Biocompatibility refers to whether the interaction between the applied material and the organism will cause adverse immune responses or toxic reactions while being able to support the growth, proliferation, and functional expression of cells (Hennig et al., 2025). Good biocompatibility is a key prerequisite for the application of hydrogels in the biomedical field. Temperature-sensitive hydrogels can interact with cells and tissues *in vivo* and can be gradually degraded *in vivo* without causing obvious immune responses and cytotoxicity (Pandya et al., 2025), avoiding the potential risks brought by long-term implantation. Meanwhile, the three-dimensional network structure of hydrogels can provide a suitable microenvironment for chondrocytes or mesenchymal stem cells, etc., promoting cell adhesion, proliferation, differentiation, and metabolism. This can also enable the temperature-sensitive hydrogel to regulate the inflammatory response and reduce tissue damage during the process of cartilage injury repair. It has been proved through *in vivo* and *in vitro* experiments on rats that an injectable thermosensitive chitosan hydrogel has good biocompatibility and biodegradability (Alyeh et al., 2023; Westin et al., 2020), and such materials are often used in the regeneration and repair of bones, cartilage, hearts, wounds, and other diseases.

### 2.3 Controllability

Thermosensitive hydrogels not only have adjustable temperature but also play a role in precisely regulating aspects such as gelation time, mechanical properties, solubility and swelling, degradation rate, controlled drug release, and microenvironment (Hanafy and El-Ganainy, 2020). Their behavior can be regulated by changing the composition, structure, and preparation process of the material. For example, the performance of hydrogels can be optimized by adjusting the length of the polymer chains and the crosslinking density and introducing specific functional groups. Stable molecular chains and more crosslinking sites can increase the crosslinking degree of hydrogels, promote higher compressive strength, and regulate the mechanical properties and swelling properties of hydrogels (Huang C. J. et al., 2024). At the same time, the gel can carry targeted treatments such as drugs, ions, and stem cells to meet the repair needs of different articular cartilage injuries.

The regulation of mechanical properties of temperature-sensitive hydrogels is the key to their adaptation to cartilage repair, and the compressive modulus and dynamic viscoelasticity of natural articular cartilage are the core of its mechanical characteristics (Venkata and Ravi, 2023). Natural articular cartilage can withstand repeated cyclic stress during the gait cycle, while thermosensitive hydrogels repeatedly rub and squeeze in the joints, causing fatigue wear or modulus decrease, resulting in structural collapse (Sergi et al., 2024). Moreover, after long-term immersion in joint fluid, the lubricating performance is significantly reduced. In future research, the mechanical stability should be improved through topological structure optimization to enable the hydrogel to exert a lasting effect and be regulated in real time according to the damage condition and *in vivo* regulation.

## 3 The application of thermosensitive hydrogel in the repair of articular cartilage injury

### 3.1 Cell carrier

Thermosensitive hydrogels can be used as cell carriers to load and encapsulate seed cells such as chondrocytes or bone marrow mesenchymal stem cells (BMMSCs) into a three-dimensional hydrogel network and deliver them to the site of articular cartilage injury by injection (Jiang J. et al., 2025; Xiao et al., 2025), making them more targeted, controllable, and intelligent. At the liquid precursor stage, where the temperature is lower than the LCST, the hydrogel presents a low-viscosity sol state. Its molecular chains are highly hydrated with water molecules through hydrogen bonds, giving Newtonian fluid-like properties. The surface tension of the sol state is low, and it has high compatibility with the phospholipid bilayer of the cell membrane. Cells can be dispersed into a single-cell suspension by vortex or micropipette (Suljovrujic et al., 2023). The material itself carries a negative charge. For example, the potential of chitosan is approximately −25 mV. Through electrostatic repulsion, it prevents cell aggregation and sedimentation. At the same time, the osmotic pressure matches the cytoplasm to avoid osmotic stress (Liu H. Y. et al., 2025). When the temperature rises above the LCST, the molecular conformation changes, and the hydrophobic groups of the polymer dehydrate to form physical crosslinking points, establishing a three-dimensional network (In and Crespy, 2025). During the gelation process, the hydrated layer gradually contracts to generate microchannels of approximately 1–5μm, guiding the cells to the center of the pores through fluid shear force (Li L. et al., 2025). The result is the formation of a gel that is close to the ECM of cartilage and has a viscoelastic modulus, which not only limits cell displacement (mobility <5 μm/h) but also allows nutrient diffusion (Ks et al., 2025; Deng et al., 2023). Hydrogels can provide a scaffold protection for cells, preventing them from being damaged during the injection process, and at the same time, offer a suitable microenvironment for cell growth and differentiation. At body temperature, hydrogels change from liquid to gel state, fixing cells at the damaged site and promoting cell proliferation and differentiation. The hydrogel can be implanted into the joint cavity by injection, rapidly forming a gel in the body to fill the cartilage defect area, avoiding complex surgical operations and the problem of implant fixation. Studies have shown that after the thermosensitive hydrogel loaded with chondrocytes is injected into the cartilage defect model, the cells can adhere and proliferate within the gel, secrete extracellular matrix, inhibit cartilage degradation and inflammatory responses, and promote the repair of cartilage tissue. In addition, MSCs have multi-directional differentiation potential and can secrete COLⅡ and various cytokines (Zhang B. K. et al., 2025), such as chemokines, growth factors, colony-stimulating factors, tumor necrosis factors, and interferons. Under the appropriate microenvironment, they can differentiate into chondrocytes and promote the expression of cartilage-specific genes. The combination of mesenchymal stem cells and temperature-sensitive hydrogels can achieve more effective repair of articular cartilage injuries.

### 3.2 Delivery system

**FIGURE 3:**
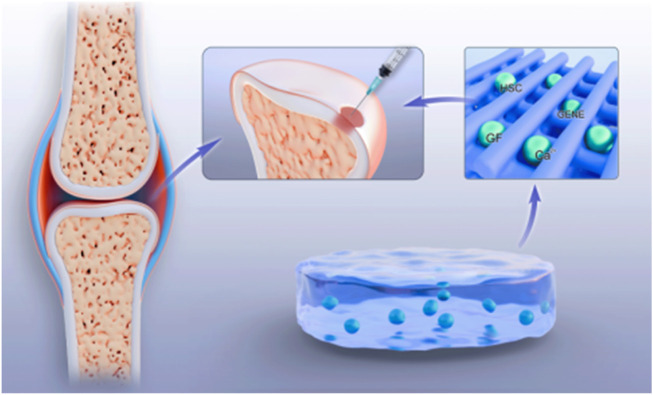
Hydrogels carry drugs, ions, etc.

The repair process of articular cartilage injury requires the participation of various growth factors and drugs to promote cell proliferation, differentiation, and the synthesis of extracellular matrix. Thermosensitive hydrogels, as delivery systems, can load growth factors, anti-inflammatory drugs, etc., inside the hydrogels (Inoue et al., 2025; Liao et al., 2024) (Figure 3). Anggelia et al. (2024) demonstrated that materials such as pomeroxam, collagen, and PLGA, as carriers, can effectively maintain the structural integrity of drugs, hormones, and growth factors as delivery systems in animal models. When the ambient temperature is low, the release of drugs is mainly driven by the concentration difference between the internal and external solutions of the hydrogel. As the temperature rises, the hydrogen bonds inside the hydrogel break and the network structure collapses, promoting the release of the carried factors, drugs, etc. (Duan et al., 2024). By controlling the release rate and amount, with the slow degradation of the hydrogel, drugs or bioactive substances such as growth factors and genes are gradually released (Yi H. Y. et al., 2023), continuously exerting their effects to achieve precise and continuous treatment of the injured areas of articular cartilage. This drug delivery method avoids the risks of infection and pain caused by multiple injections and greatly reduces the economic burden on patients.

Shi et al. (2021) inserted circRNA3503 loaded with a temperature-sensitive hydrogel, which could effectively promote the proliferation and migration of chondrocytes and simultaneously inhibit the inflammatory response, into the site of a cartilage injury. The research team of Alizadeh et al. (2025) loaded transforming growth factor -β3 (TGF-β3) or recombinant human bone morphogenetic protein-2 (rhBMP-2) into temperature-sensitive hydrogels, which could form local high-concentration areas at the injury site and be slowly released over several weeks, stimulating the proliferation, differentiation, and extracellular matrix synthesis of chondrocytes. Meanwhile, the sustained release of drugs can also reduce the dosage of drugs and systemic side effects, improve the safety and effectiveness of treatment, and significantly promote cartilage repair. Thermosensitive hydrogels are helpful in reducing the inflammatory response within joints (Han et al., 2025; Liu H. et al., 2025). Tian et al. (2024) confirmed through experiments on rats that thermosensitive hydrogels have antibacterial properties, achieving an antibacterial rate against *Escherichia coli* of 87.17% ± 2.48%, which was significantly higher than the 54.41% ± 4.29% rate of the control group. Thermosensitive hydrogels can also reduce the catabolism of chondrocytes under inflammatory conditions, inhibit inflammatory responses, and create a relatively stable internal environment for cartilage repair.

### 3.3 Cartilage tissue engineering scaffold

By adjusting the material composition and crosslinking degree, temperature-sensitive hydrogels serve as tissue engineering scaffolds (Ren et al., 2025) and simulate the extracellular matrix of natural cartilage as well as its mechanical properties and biological activities using a three-dimensional network structure to provide support for cell growth and tissue construction. After being combined with chondrocytes or bone marrow mesenchymal stem cells, the material is implanted into the injured area of articular cartilage. Especially for irregular injuries, the effect of restoring the integrity and smoothness of the joint surface is better. The temperature-sensitive hydrogel scaffold provides a favorable growth environment for cells, promotes stem cell recruitment and cartilage differentiation, and maintains the original structure and function of cartilage tissue at the same time. The mechanical properties of the scaffold can also be further enhanced by adding bioactive molecules and nanomaterials, such as hydroxyapatite (Jahangir et al., 2023), to activate the biological activity and promote the differentiation and proliferation of chondrocytes (Zigan et al., 2024). With the development of 3D printing technology, personalized hydrogel scaffolds can be precisely prepared based on the shape and area of articular cartilage injuries, achieving more accurate repair and treatment.

At present, the most common temperature-sensitive hydrogels include blocks of PNIPAM (Qian et al., 2024) and polyethylene glycol (PEG), CS (Fan et al., 2025) hydrogels, sodium hyaluronate hydrogels, polyoxide–polyoxide–polyethylene oxide (PEO-PO-PEO) hydrogels, stereocomposite polylactic acid temperature-sensitive hydrogels, etc. Among them, the most common ones are PNIPAM and PEG blocks (Gao et al., 2021; Zhang X. Y. et al., 2025). PNIPAM is a heat-sensitive polymer material that has been used in some studies for the repair of articular cartilage. Its temperature-sensitive properties contribute to good operation and adaptation to the *in vivo* environment. Mixing chondrocytes or BMMSCs with PNIPAM solution and injecting them into the cartilage defect site can trigger gelation at body temperature (37.0°C), fix the cells, and form a porous three-dimensional scaffold, which helps reduce the load on the joint and minimize secondary injuries. PEG is temperature-sensitive and degradable. The degradation rate can be regulated by adjusting the molecular weight (Liu and Zhou, 2025). It can provide mechanical support in the early stage of injection and implantation injury site and is gradually replaced by extracellular matrix in the later stage, matching the degradation rate with the cartilage regeneration rate. Moreover, through the relaxation characteristics of hydrogel, it promotes the conduction of mechanical signals to seed cells and accelerates injury repair. CS hydrogel utilizes its natural antibacterial properties to prevent infection (Tu et al., 2024), and, at the same time, loads BMMSCs to promote cartilage regeneration.

Complex exosomes or miRNAs regulate the inflammatory microenvironment and promote cartilage differentiation through a sustained-release gel system. It is possible to form a pH/temperature dual-responsive gel by introducing temperature-sensitive components such as sodium β -glycerophosphate (β-GP) (Pham et al., 2025). Jiang et al. (2024) added magnesium particles (MPs) to glycerophosphate solution and mixed it with CS solution to prepare porous injectable thermosensitive hydrogels containing magnesium ions. The incorporation of MPs formed interconnected pores in the hydrogel, demonstrating high cytocompatibility and maintaining cell viability, proliferation, diffusion, and osteogenesis *in vitro*. The pore size can allow the migration, proliferation, and differentiation of cells in the hydrogel scaffold. However, an increase in porosity will reduce the mechanical properties of the scaffold, while insufficient porosity will lead to poor regeneration (Mo et al., 2021). Sodium hyaluronate hydrogel is the sodium salt form of hyaluronic acid. As a natural component of human joint fluid and cartilage, it has excellent biocompatibility and degradability, reducing the risk of immune rejection (Hanyu et al., 2024). A three-dimensional network structure is formed through physical crosslinking, with a high water content of approximately 74 wt%, simulating cartilage ECM and providing a suitable microenvironment for cells (Zhou et al., 2025). The gel interface can enhance the combination with the surrounding cartilage tissue through physical crosslinking, reducing the risk of detachment (Xiang et al., 2024; Li G. F. et al., 2024). Hydrogels can enhance the viscoelasticity of joint fluid, improve joint range of motion, relieve pain, and promote cartilage repair through multi-dimensional mechanisms (Steale et al., 2025). PEO-PPO-PEO hydrogel is a temperature-sensitive triblock copolymer that can form a physically cross-linked gel at body temperature, providing a three-dimensional porous structure similar to that of natural cartilage ECM and promoting the adhesion, proliferation, and differentiation of chondrocytes. The inflammatory microenvironment of the joint cavity can be alleviated by loading anti-inflammatory drugs or directly regulating the polarization of macrophages through the surface properties of the materials. Inhibit the excessive proliferation of fibroblasts, reduce the formation of scar tissue (Zhang F. et al., 2025), promote the regeneration of hyaline cartilage rather than fibrocartilage, and avoid the recurrence of fibrocartilage fragmentation during joint movement. By adjusting the molecular weight and proportion of the copolymer, such as the PEO/PPO ratio, the mechanical strength can be regulated to simulate the elasticity and compressive resistance of cartilage and provide temporary mechanical support for the damaged area (Rick, 2021). The steric composite polylactic acid (sc-PLA) thermosensitive hydrogel is formed by the intermolecular hydrogen bonds of L-polylactic acid (PLLA) and d-polylactic acid (PDLA) to create a steric composite structure. It has a higher melting point (220°C), mechanical strength (modulus up to 10 GPa), and hydrolysis resistance (Park et al., 2025). The degradation rate can be regulated by the three-dimensional composite ratio to avoid the problem of too fast or too slow degradation of a single PLA and better match the cartilage regeneration cycle. It also promotes cartilage regeneration through the synergy of multiple mechanisms such as mechanical support, cell delivery, and factor sustained release.

### 3.4 Unique effects at the cellular and tissue levels

#### 3.4.1 Cellular level

Thermosensitive hydrogels, such as PNIPAM, are liquid at low temperatures and can be uniformly mixed with cells. When the temperature rises to the physiological temperature, a gel network is rapidly formed, enveloping the cells in a three-dimensional structure. The cell survival rate is high (Thanh et al., 2024). The enveloping process does not require ultraviolet light or chemical crosslinking, reducing cell stress and toxic damage to the cells, achieving mild cell enveloping and protection (Constantin et al., 2025). By adjusting the hydrophilicity, hydrophobicity, and porosity of temperature-sensitive hydrogels, the exposure of cell adhesion proteins (such as fibronectin and laminin) can be controlled, affecting cell adhesion, migration, and proliferation. By altering the temperature to induce changes in gel strength (Li Y. F. et al., 2024), the mechanical microenvironment of stem cells can be regulated, guiding their differentiation toward specific lineages, such as osteogenic and cartilaginous differentiation, and dynamically regulating cell behavior. Thermosensitive hydrogels can be used as sustained-release carriers to encapsulate growth factors (such as VEGF or TGFβ) or small-molecule drugs (Liu Q. Y. et al., 2025). By triggering the swelling/contraction of the gel through temperature changes, the drug is released on demand, and the cellular signaling pathways are precisely regulated. At the same time, the material can provide a bionic three-dimensional microenvironment, support intercellular interactions and organoid formation, and realistically simulate the physiological conditions *in vivo*.

#### 3.4.2 Organizational level

Thermosensitive hydrogels can be directly injected into the body by injecting liquid precursors at the site of cartilage defects. They can form gels *in situ* at body temperature and fill irregular tissue defects without surgical intervention. The mechanical properties, such as the elastic modulus and pore structure of hydrogels, can simulate natural ECM, promoting cell infiltration and tissue regeneration. When there is often an increase in local temperature at the site of inflammation or lesion, thermosensitive hydrogels can release anti-inflammatory drugs or recruit repair cells, such as MSCs, through temperature response to achieve intelligent responsive treatment (Bucatariu et al., 2024). Moreover, by regulating the degradation rate, angiogenic factors can be gradually released to promote the growth of new blood vessels in the soft tissues within the joint (Yang et al., 2024), increase local blood circulation, and facilitate the rapid repair of combined injuries. Temperature-sensitive hydrogels exhibit unique regulatory effects at the cellular and tissue levels through the construction of dynamic bionic microenvironments that are distinct from traditional scaffolds.

### 3.5 Combined application

Temperature-sensitive hydrogels can also be combined with other biomaterials or therapeutic methods (Gao et al., 2025). For example, in some special injuries or severe traumas that cause irregular or large-area defects of articular cartilage, traditional drilling surgery, microfracture surgery, and cartilage transplantation surgery combined with temperature-sensitive hydrogels can be used for treatment to make up for each other’s deficiencies and maximize the therapeutic effect. It is also possible to combine and adapt various repair materials that promote the proliferation and differentiation of chondrocytes (Qian et al., 2025) or carry multiple therapeutic drugs and factors in the same hydrogel. Sobrino et al. (2024a) successfully transplanted and re-cultured osteoblast membranes rich in type I collagen using nanoparticle technology loaded with icariin, maintaining the optimal ECM composition and promoting the repair of cartilage damage. The material can also be combined with nanomaterials, conductive polymers, or bioactive glass composites (SiCong et al., 2024) to enhance mechanical properties, electrical activity, or osteogenic ability and jointly exert therapeutic effects. The hydrogel can be made to fit the missing area perfectly, like natural cartilage, and maximize the repair effect.

At present, bilayer hydrogels with bionic mechanical gradients are being developed (Zhang T. et al., 2025), such as the combination of a surface highly cross-linked PLGA layer and a bottom temperature-sensitive gelatin layer, which greatly enhances the interfacial shear strength. The RGD peptide and TGF-β1 microarray were fixed in the interface area by photografting technology, which significantly increased the migration rate of chondrocytes (K et al., 2020). Polypyrrole nanowires can also be doped to generate a local electric field through the piezoelectric effect (Li M. et al., 2024), promoting the vertical directional growth of COLII fibers, achieving precise integration of biological activity and electrical activity, and facilitating rapid repair of damaged areas (Du et al., 2024). Kang et al. (2023) used an oxygenated thermosensitive hydrogel containing 1-bromosefluorooctane in the repair of femoral condylar bone defects in a rabbit experiment. It effectively promoted the formation of new bone, increased tissue vascular differentiation at the same time, and was more conducive to the repair of surface cartilage injury Table 3.

**TABLE 3:** Summary of applications of temperature-sensitive hydrogel in repairing articular cartilage injury.

Application	Specific use	Shortcomings	Literature source
Cell carrier	Cells are loaded in the three-dimensional network of the hydrogel, providing a suitable microenvironment for the growth and differentiation of cells. Promote cell adhesion and proliferation, secrete extracellular matrix, and facilitate the repair of cartilage tissue.	According to the changes in body temperature, if the phase transition is not timely, the loaded cells will be compressed and damaged, or they will not be released sufficiently at the designated site, or they will be released too early. The image restoration effect will be poor.	Sobrino et al. (2024b), Sobrino et al. (2024c)
Delivery system	Achieve temperature-controlled release of drugs or growth factors, etc.	The hydrophilic solubilization effect is inconsistent, the size of the scaffold voids is not uniform, and the drugs, ions, and genes carried lose their normal sustained-release speed and efficiency.	Sun et al. (2024b), J et al. (2023)
Cartilage tissue engineering scaffold	The stability of mechanical properties is uncertain depending on temperature changes. During the degradation process of the temperature-sensitive hydrogel scaffold, the immune response and toxic side effects cannot be removed.	The stability of mechanical properties is uncertain depending on temperature changes. During the degradation process of the temperature-sensitive hydrogel scaffold, the immune response and toxic side effects cannot be removed.	Mo et al. (2021), Li et al. (2025b)
Combined application	Thermosensitive hydrogels can also be used in combination with other biomaterials or therapeutic methods to maximize their therapeutic effects.	The preparation process is complex, and the stability is insufficient.	Kang et al. (2023), Huang et al. (2024b)

## 4 The shortcomings of temperature-sensitive hydrogels

In practical applications, temperature-sensitive hydrogels have many deficiencies that need to be further optimized. In terms of temperature control, it is difficult to precisely control the phase transition temperature of temperature-sensitive hydrogels based on LCST, especially in deep tissues, in high-cold regions, or under pathological conditions such as inflammation and trauma. The body temperature changes due to the different degrees of blood flow richness in local areas, causing the gel cure to be delayed or premature, resulting in a mismatch of gel morphology in the defect area (Colombo et al., 2025). The thermal conductivities of different tissues (such as subchondral bone, synovium, and fat pads) vary. After injection, the hydrogel may form “cold zones” or “hot zones” due to the differences in thermal conduction of the surrounding tissues, resulting in uneven porosity or crosslinking density within the gel (Wang et al., 2025; Li D. P. et al., 2025). Deep tissues (such as acetabular cartilage defects) have a lower temperature than superficial tissues (such as knee cartilage), and gradient LCST materials must be designed to meet the requirements of different depths (Stanzione et al., 2024).

In terms of drug sustained release, the hydrophilic swelling performance of hydrogels varies, and the internal circulation and water content within the joints of different populations also differ, resulting in uneven swelling effects. This leads to different pore sizes of hydrogel scaffolds, causing the drugs, ions, and genes they carry to lose their normal sustained-release speed and efficiency (Guo et al., 2023). The PNIPAM thermosensitive hydrogel still has disadvantages such as a low swelling ratio, a small re-swelling rate, and poor mechanical strength (Jiang et al., 2025c). In terms of degradation, the mismatch between the degradation rate of the hydrogel and the tissue regeneration rate during the degradation process, as well as the degradation products generated, increase the immune response during the absorption process (Li M. Y. et al., 2023).

Although chitosan (CS), a natural polysaccharide, has good biocompatibility, when its concentration is too high, its degradation product glucosamine can be metabolized into urea and carbon dioxide in the body, which may cause an increase in the secretion of TNF-α by macrophages and lead to a local mild inflammatory response (Pitrolino et al., 2024; Chen et al., 2025b). When the concentration of the residual unreacted monomer (N-isopropyl acrylamide) in the PNIPAM system is too high, it can significantly inhibit the gene expression of chondrocytes, and the low-molecular-weight oligomer produced by its degradation has a half-life of up to 28 days in synovial fluid, which can affect cell adhesion (Rauer et al., 2024; Komarova et al., 2025). The mechanical strength of thermosensitive hydrogels still lags behind that of natural cartilage. The persistent friction and compression caused by daily activities such as walking, running, and jumping are difficult to withstand, and joints are under complex stress, resulting in fatigued fragmentation of hydrogels or immune responses, etc. (Liu et al., 2020a; Liu et al., 2020b). Existing studies have fully verified the advantages of temperature-sensitive hydrogels in terms of injectability, minimal invasiveness, and the construction of dynamic microenvironments (Khan et al., 2024). However, although rapid gelation is beneficial for morphological fixation, the high shear force environment leads to a decrease in the survival rate of MSCs. Enhancing mechanical properties is often accompanied by an prolonged degradation cycle, which is inconsistent with the matching time of the cartilage regeneration cycle (Hu et al., 2025; Wang J. S. et al., 2024). The increase in the encapsulation rate of growth factors instead leads to a decrease in biological activity, reducing the accuracy of controlled drug release and bioavailability.

In response to these issues, in future research, we still need to verify and improve the deficiencies of temperature-sensitive hydrogels through more animal experiments and clinical trials so as to enhance their mechanical properties, histocompatibility, and biodegradability; maintain the stability of temperature-sensitive hydrogels under the physiological and pathological conditions of the organism; and fully exert their good biological efficacy. Genetic engineering technology can be combined to develop intelligent hydrogels with intrinsic biological activities that enable them to play a greater role in the repair of articular cartilage.

## 5 Summary and prospect

Temperature-sensitive hydrogels, which are intelligent biomaterials, have shown great potential in the repair of articular cartilage injuries. They share commonalities with other hydrogels and have their own characteristics. Their unique temperature response characteristics, good biocompatibility, and controllable degradation performance can be used for filling, protecting, and lubricating scaffolds and material exchange at cartilage injury sites. The sol–gel transformation occurs at body temperature, forming a gel scaffold with a three-dimensional network structure, providing a favorable growth microenvironment for chondrocytes and promoting cell proliferation, differentiation, migration, and matrix secretion. They can simultaneously carry cells, drugs, genes, growth factors, etc. Changing the pore size and gel phase of the hydrogel scaffold adjusts the release rate of these carried materials, achieving good *in situ* retention and efficient delivery, and improving cell survival rate, drug delivery efficiency, and repair effect.

Based on its injectable and *in situ* gelation characteristics, through minimally invasive injection assisted by arthroscopy, precise filling of cartilage defects in complex anatomical sites, such as the posterior condyle of the knee joint, the acetabular fossa, and the ankle joint, can be achieved in the future. Combined with medical image navigation techniques such as intraoperative ultrasound or fluorescence labeling, the distribution and gelation state of hydrogels at the defect site can be monitored in real time to ensure the anatomical matching degree between the three-dimensional morphology and natural cartilage. Patient-specific temperature-sensitive hydrogel stents have been prepared by 3D printing technology. The three-dimensional model of the defect was reconstructed using preoperative CT/MRI data, and personalized stents with bionic gradient pores and mechanical gradients were designed.

In time, a treatment team will construct a multimodal combined treatment plan, establish a “hydrogel + clinical” plan, combine shock wave therapy, and enhance the penetration depth of growth factors in the hydrogel through a cavitation effect. Combined with methods such as gene editing, the gene expression is continuously upregulated in the defect area to promote the synthesis of COLII. Through interdisciplinary collaboration and technological innovation, temperature-sensitive hydrogels are expected to become standardized treatment plans for cartilage repair, promoting the transformation of regenerative medicine from the model of “structural substitution” to that of “functional reconstruction.”

With the continuous cross-integration of multiple disciplines such as materials science and biomedical engineering, temperature-sensitive types have broad application prospects in cartilage regeneration. Future research should focus on the following aspects: First, develop new temperature-sensitive materials with a wider temperature response range, faster response speed, and better biocompatibility, and conduct in-depth studies on the relationship between the microstructure and performance of hydrogels to better understand their temperature response mechanism and optimize their performance. Research simpler and more efficient hydrogel preparation methods and more precise characterization techniques to meet the needs of damage repair in different parts of the body. Second, conduct in-depth research on the interaction mechanism between hydrogels and mesenchymal stem cells and tissues, optimizing the loading mode of cells and drugs, and improving the repair effect. Third, strengthen basic research and clinical trials to promote the early clinical transformation and wide application of temperature-sensitive hydrogels. At the same time, enhance research on special injury types such as epiphyseal injuries in children and injuries at the cartilage-bone junction. Fourth, multiple stimulus response factors such as temperature, pH value, light, and enzymes can be combined to develop dual-sensitive or multi-sensitive response materials with multiple functionalization. The fifth research focus is to develop mature temperature-sensitive hydrogel products with independent packaging that can be directly applied in clinical practice. With the continuous development of 4D printing technology and artificial intelligence (AI) technology, AI technology may be applied to precisely regulate the composition and parameter adjustment of temperature-sensitive hydrogels, establish a multi-scale evaluation system, and integrate molecular dynamics simulation, organ-chips, and AI-assisted clinical decision-making systems. Develop new types of hydrogels with body temperature feedback functions, such as integrating temperature sensors, to achieve real-time temperature regulation. These would enable real-time monitoring and dynamic adjustment during the process of cartilage injury repair, reducing treatment costs, shortening treatment time, and efficiently utilizing thermosensitive hydrogels. Through continuous improvement and optimization, the hydrogel can play a greater role in the repair of cartilage damage and approach the performance of natural cartilage to the greatest extent. These characteristics make hydrogels an ideal repair material for cartilage injury repair that could be widely applied.
